# Association of small fiber neuropathy and post treatment Lyme disease syndrome

**DOI:** 10.1371/journal.pone.0212222

**Published:** 2019-02-12

**Authors:** Peter Novak, Donna Felsenstein, Charlotte Mao, Nadlyne R. Octavien, Nevena Zubcevik

**Affiliations:** 1 Department of Neurology, Brigham and Women’s Hospital, Harvard Medical School, Boston, Massachusetts, United States of America; 2 Department of Infectious Disease, Massachusetts General Hospital, Harvard Medical School, Boston, Massachusetts, United States of America; 3 Dean Center for Tick Borne Illness, Spaulding Rehabilitation Hospital, Boston, Massachusetts, United States of America; 4 Department of Physical Medicine and Rehabilitation, Spaulding Rehabilitation Hospital, Boston, Massachusetts, United States of America; 5 Dean Center for Tick Borne Illness, Department of Physical Medicine and Rehabilitation, Spaulding Rehabilitation Hospital, Harvard Medical School, Boston, Massachusetts, United States of America; Weill Cornell Medicine-Qatar, QATAR

## Abstract

**Objectives:**

To examine whether post-treatment Lyme disease syndrome (PTLDS) defined by fatigue, cognitive complaints and widespread pain following the treatment of Lyme disease is associated with small fiber neuropathy (SFN) manifesting as autonomic and sensory dysfunction.

**Methods:**

This single center, retrospective study evaluated subjects with PTLDS. Skin biopsies for assessment of epidermal nerve fiber density (ENFD), sweat gland nerve fiber density (SGNFD) and functional autonomic testing (deep breathing, Valsalva maneuver and tilt test) were performed to assess SFN, severity of dysautonomia and cerebral blood flow abnormalities. Heart rate, end tidal CO_2_, blood pressure, and cerebral blood flow velocity (CBFv) from middle cerebral artery using transcranial Doppler were monitored.

**Results:**

10 participants, 5/5 women/men, age 51.3 ± 14.7 years, BMI 27.6 ± 7.3 were analyzed. All participants were positive for Lyme infection by CDC criteria. At least one skin biopsy was abnormal in all ten participants. Abnormal ENFD was found in 9 participants, abnormal SGNFD in 5 participants, and both abnormal ENFD and SGNFD were detected in 4 participants. Parasympathetic failure was found in 7 participants and mild or moderate sympathetic adrenergic failure in all participants. Abnormal total CBFv score was found in all ten participants. Low orthostatic CBFv was found in 7 participants, three additional participants had abnormally reduced supine CBFv.

**Conclusions:**

SFN appears to be associated with PTLDS and may be responsible for certain sensory symptoms. In addition, dysautonomia related to SFN and abnormal CBFv also seem to be linked to PTLDS. Reduced orthostatic CBFv can be associated with cerebral hypoperfusion and may lead to cognitive dysfunction. Autonomic failure detected in PTLDS is mild to moderate. SFN evaluation may be useful in PTLDS.

## Introduction

Lyme disease is a transmittable tick-borne infection caused by the spirochete Borrelia burgdorferi [1–3]. Lyme disease is a serious public health problem. It is estimated that approximately 300,000 cases occur annually in the US [2]. The reported annual incidence ranges from 20–100 cases per 100,000 people in endemic areas.

Neurologic sequelae of Lyme disease, termed Lyme neuroborreliosis, occurs in 10–15% of patients with untreated Lyme disease [2,4,5]. The spirochete can invade peripheral and central nervous system and may cause aseptic meningitis, cranial neuropathy, painful radiculitis or encephalo-myelo-radiculitis and various forms of peripheral neuritis.

Persistent symptoms despite standard antibiotic therapy of Lyme disease are reported in 10% to 36% of patients [6–11]. These symptoms, when prolonged for a period of 6 months or longer, are referred to as post-treatment Lyme disease syndrome (PTLDS). Typical symptoms of PTLDS include widespread pain, fatigue, and cognitive disturbances. There is considerable impairment of health-related quality of life among patients with PTLDS.

The origin of PTLDS symptoms is unclear [7,10]. Potential mechanisms include direct cytotoxicity by the spirochete, neuroinflammation or autoimmune reactions. These potential mechanisms may cause damage to the central and peripheral nervous systems and be one of the reasons for life-altering symptoms of fatigue, widespread pain and cognitive deficits experienced by patients with PTLDS.

The main problem in PTLDS research is lack of an objective biomarker [7]. We hypothesize that some of the sensory symptoms in PTLDS are due to small fiber neuropathy (SFN). To establish whether SFN might serve as an objective biomarker of PTLDS, we designed this study to evaluate associations between PTLDS and SFN using skin biopsies that provide direct evidence of small fiber damage. Secondary aims were evaluations of autonomic dysfunction associated with presumed SFN and assessment of cerebral blood flow since cognitive complaints may be due to cerebral hypoperfusion.

## Materials and methods

This retrospective, single-center study included consecutive patients with history of PTLDS who underwent autonomic testing between 2016 and 2018 at the Brigham and Women’s Faulkner Hospital Autonomic laboratory.

### Clinical definitions

PTLDS was defined following the Aucott’s criteria [7] which include: 1) combination of fatigue, cognitive complaints and chronic widespread pain following the treatment of Lyme disease for at least 6-months period; 2) absence of other disorder that can explain the complaints associated with PTLDS; 3) documented history of Lyme disease satisfying the CDC criteria [12,13].

In this study Lyme disease was defined as: (1) a history of symptoms indicative of Lyme disease; (2) positive serology, Lyme IgG western blot, per CDC criteria [12,14,15]. The IgG immunoblot was considered to be positive with the presence of 5 or more of the following bands: 18, 23 [OspC], 28, 30, 39, 41, 45, 58, 66, and 93 kDa IgG bands.

Since all patients had symptoms longer than 30 days, IgM immunoblot results, which are part of CDC criteria for acute (<4 weeks) Lyme disease, were not taken into consideration for purposes of Lyme disease definition in this study.

Inclusion criteria for this study were: (1) patients older than 17 years of age; (2) history of Lyme disease as defined above; (3) persistence of continuous or relapsing symptoms for greater than 6 months after completing antibiotic therapy; (4) evaluation for SFN and related dysautonomia at the Autonomic Laboratory at Brigham and Women’s Faulkner Hospital; (5) availability of medical records.

The exclusion criteria for the study were: (1) disorders associated with secondary SFN; (2) the use of medication that affect autonomic functions, including anticholinergic medication and medication for treatment of hypertension or hypotension; (3) inability to complete the autonomic testing; (4) missing skin biopsy results; (5) large fiber neuropathy.

Medical records were reviewed for secondary causes of SFN which include diabetes, borderline diabetes, large fiber neuropathy, Parkinson's disease, atypical parkinsonism, alcohol abuse, B12 deficiency, folate deficiency, thyroid disease, celiac disease, hepatitis C, HIV infection, exposure to chemotherapy, cancer, any comorbid conditions or use of medication reported to be associated with small fiber neuropathy [16–18]. The medical records were carefully reviewed for evidence of large fiber damage including loss of tendon reflexes, weakness, and large-fibers mediated sensory loss (proprioception and vibration sensation) on the neurologic examination.

### Standard protocol approvals, registrations, and patient consents

The Institutional Review Board of the Brigham and Women’s Hospital, Harvard University approved the study.

### Autonomic tests

Standardized autonomic testing included deep breathing, the Valsalva maneuver, the tilt test and sudomotor evaluation; and was described in details previously [16,19–22]. The following signals were recorded: electrocardiogram, continuous and intermittent blood pressure, end tidal CO_2_ and cerebral blood flow velocity (CBFv) in the middle cerebral artery using transcranial Doppler. Sudomotor testing was done using the quantitative sudomotor axon reflex test [23] or using electrochemical skin conductance (ESC) [16]. Normative data for ESC adjusted for weight are hands ≥1.03 μS/kg and feet ≥1.14 μS/kg [22].

Normative supine data for CBFv age and gender- dependent. Lower limit for women/men are 82.2–0.45 (cm/s) * age (years) /72.09–0.38 (cm/s) * age (years) [20]. In healthy subjects, orthostatic CBFv is either unchanged or may slightly decline. The normal decline in orthostatic CBFv is equal or less than 10% (1th minute), 11% (5th minute) and 15% (10th minute) of the tilt baseline [20].

During the testing, all subjects were assessed for the presence of sensory complaints using the Neuropathy Total Symptom Score-6 [24] and autonomic symptoms using the Survey of Autonomic Symptoms [25].

### Skin biopsies

Skin biopsies for assessment of ENFD and SGNFD were obtained from the right calf (10 cm above the lateral malleolus) using a 3-mm circular disposable punch tool using established standards [26,27]. All processing was done at Therapath (New York, NY). Details of sample fixation, processing and fiber counting were described previously [16]. Shortly, skin samples were placed into 2% paraformaldehyde lysine periodic acid fixative, then immunoperoxidase stained with axonal marker PGP 9.5 and ENFD/SGNFD were counted using light microscopy [27,28]. We used the University of Massachusetts’s age- and gender- adjusted normative value for ENFD (fibers per millimeter of epidermal length) at the calf which are equal to 9.5–0.075 * age for men and 11.1–0.08 * age for women) [20]. The normative data for SGNFD (≥36.5 fibers per millimeter at the calf) were obtained from Therapath.

### Grading of results

The tests results were graded using Quantitative Scale for Grading of Cardiovascular Reflex Tests, Transcranial Doppler, Quantitative Sudomotor Axon Reflex Test, and Small Fiber (epidermal sensory and sweat gland) Densities from Skin Biopsies (QASAT) [20]. QASAT is the validated objective instrument for grading of dysautonomia, related small fiber neuropathy, and cerebral blood flow. QASAT assigns scores to test results, where 0 is normal, and above 0 is abnormal. Abnormal scores are further stratified into mild, moderate, severe, abnormality or market abnormalities. For example the numeric scores for ENFD are 1 (mild abnormality), 2 (moderate abnormality), 3 (severe abnormality) and 4 (market abnormality). Tilt-related scores have wider dynamic range, for example orthostatic hypotension score has range 0–10.(normal = 0, mildly abnormal = 1,2; moderately abnormal = 2,3, severely abnormal = 5,6; markedly abnormal >6).

## Statistical analysis

Descriptive statistic was used to assess the presence of SFN and autonomic dysfunction. JMP 12 (Cary, NC, USA) statistical software was used for statistical analyses.

## Results

We screened 133 participants with suspected history of Lyme disease that were referred for evaluation of autonomic functions. Eleven participants satisfied the CDC criteria of Lyme disease and the Aucott’s criteria for PTLDS. One participant was excluded from the study since autonomic testing was not completed for technical reasons. Ten participants, 5/5 women/men, age 51.3 ± 14.7 years, BMI 27.6 ± 7.3 and were included in the final analysis (Table 1).

**Table 1:** Basic demographic of patients with PLDS with results of the immunoblot for Lyme disease.

No	Age	G	BMI	DD	IgG band												
					18	23	26	28	30	39	41	45	47	58	61	66	93
1	63	F	31.4	10	+	+				+	+	+				+	+
2	65	M	42.7	5	+	+				+	+	+				+	
3	60	M	37.5	10	+	+		+		+	+	+		+		+	+
4	23	M	21.3	6	+	+					+					+	+
5	57	F	22.3	6	+					+	+			+			+
6	32	F	23.7	3		+				+	+	+				+	
7	44	M	25.9	9	+		+		+		+		+	+	+		
8	53	F	23.4	4	+	+		+	+	+	+	+		+		+	+
9	68	F	21.6	17		+		+	+		+			+			
10	48	M	1061	4	+	+		+		+	+					+	+

No, the participant number; G,gender; F, female; M, male; DD, disease or symptom’s duration in years.

Neurological evaluation in all 10 participants showed normal deep tendon reflexes, normal vibration and proprioception sense, the modalities transmitted by large fibers [29]. Nerve conduction studies were performed in 3 participants; all were normal, indicating normal large fiber functions. All patients had received a course of antibiotics for Lyme disease including 3 weeks of oral doxycycline as recommended by Infectious Diseases Society of America (IDSA) guidelines [13,30]. Because of symptoms persistence, all participants were treated with additional antibiotics (data in S1 Table). The list may not be accurate and may underestimate the antibiotic therapy as many participants were treated in multiple institutions and as such medical records may be incomplete.

The Neuropathy Total Symptom Score-6 was 8.7 ± 5.3 and the Survey of Autonomic Symptoms impact was 17.0 ± 8.6 (Table 2).

**Table 2:** Summary of symptoms.

Symptom	Number
Lightheadedness	8
Dry mouth or dry eyes	8
Pale or blue feet	6
Feet colder than the rest of body	7
Decreased sweating at feet at rest	5
Decreased sweating at feet after exercise or during hot weather	5
Sweating increased at hands	3
Nausea, vomiting or bloating after meal	2
Persistent diarrhea	4
Persistent constipation	2
Leaking of urine	3
Difficulties in erection (man)	1
Aching pain	10
Allodynia	3
Burning pain	6
Lancinating pain	6
Numbness	8
Prickling sensation	8

Number designates the number of participants with respective symptoms.

At least one skin biopsy was abnormal in all ten participants (Table 3).

Abnormal ENFD was found in 9 participants, abnormal SGNFD in 5 participants, and both abnormal ENFD and SGNFD were detected in 4 participants. Severe SFN defined as QASAT-ENFD or QASAT-SGNFD score ≥ 3 was found in 7 participants. SGNFD was not available in one participant since no sweat gland has been found on the biopsy specimen. Parasympathetic failure defined as abnormal QASAT-DB was found in seven participants. Sympathetic adrenergic failure defined as abnormal QASAT-VM + QASAT-OH was found in all participants. Orthostatic hypotension (QAASAT-OH score) was detected in one participant. Abnormal total CBFv score was found in all ten participants. Low orthostatic CBFv was found in 7 participants. Three participants without orthostatic CBFv drop had abnormally reduced supine CBFv. QASAT total score was abnormal (>0) in all participants. Detailed distribution of abnormalities is in Table 3.

**Table 3:** Results of questionnaires, skin biopsies and selected QASAT scores for each participant.

No	NTSS6	SAS	ENFD	ENFD-N	SGNFD	QASAT								
						DB	VM	OH	ENFD	SGNFD	Sudo	CBF	CO_2_	Total
1	20	22	1.15	6.06	31.1	2	2	0	4	1	4	4	2	24
2	11	13	0.27	4.625	18.7	1	2	0	4	3	3	1	0	16
3	6	5	4.57	5	15.8	0	2	0	1	4	1	4	0	12
4	3.3	26	8.31	7.775	32.4	0	0	0	0	1	1	3	0	9
5	11.7	18	4.77	6.54	50.2	2	1	0	1	0	0	3	9	18
6	13	4	5.22	8.54	N/A	0	1	0	3	N/A	0	1	0	7
7	5.33	25	3.92	6.2	63.1	2	3	0	3	0	1	3	0	13
8	7.32	8	5.05	6.86	53.9	2	0	0	1	0	0	1	3	8
9	7	22	1.37	5.66	41.2	1	0	1	3	0	1	1	2	10
10	2	27	0.08	5.9	21.5	1	2	0	4	3	1	4	4	26

No, the participant number; NTSS6, the Neuropathy Total Symptom Score-6; SAS, the Survey of Autonomic Symptoms Impact; ENFD, epidermal nerve fiber density (fibers/mm); ENFD-N, threshold of normal value, age and gender adjusted; SGNFD, sweat gland nerve fiber density (fibers/mm), normal threshold is 36.5 fibers/mm. QASAT scores: DB, deep breathing; VM, Valsalva maneuver; OH, orthostatic hypotension; ENFD, sensory small fiber neuropathy score using ENFD; SGNFD. sudomotor/autonomic small fiber neuropathy score using SGNFD; Sudo, sudomotor; CBF, total cerebral blood flow; CO_2,_ end tidal CO2.

## Discussion

There are three main finding of this study: 1) Evidence of SFN in PTLDS. Skin biopsy showed loss of small fibers in all participants. Most participants (n = 9) had evidence of sensory SFN (abnormal ENFD) and severe SFN was most common (n = 7); 2) Autonomic dysfunction was found in all PTLDS subjects. Most common was mild to moderate autonomic failure; 3); Abnormal cerebral blood flow was detected in all PTLDS participants.

The cause of SFN in our cohort remains to be clarified. We excluded participants with known secondary causes of SFN by meticulously reviewing past medical histories and disorders previously reported to be associated with SFN or peripheral nervous system dysfunction. Therefore, our observations, raise the possibility that there PTLDS and SFN are related. We believe that the interpretation that PLDS/Lyme disease and SFN are linked is plausible given abnormal serologic findings (Table 1) in our patients. Furthermore, patients with Lyme disease have been shown to have lymphoplasmocellular infiltrates in autonomic ganglia and peripheral nerves [31] which may be an underlying substrate for SFN although no spirochetes were found in respective neural structures [32].

There are several mechanisms of neuronal injury that might account for a link between Lyme disease/PLDS, and SFN. These include—direct cytotoxicity by the spirochete, the presence of neurotoxic mediators that occur during host-pathogen interaction, and triggered autoimmune reactions [33–35]. Since no spirochetes are present in patients’ specimens [31,32], inflammation, either chronic or acute, with neuropathic effect may play a role. Understanding the complex evolution from infection to inflammation may be a subject of future studies. Nevertheless, our finding yield testable hypotheses, for example that anti-inflammatory therapy may be effective in either prevention or treatment of SFN in PLDS.

This study used both morphological (skin biopsy) and functional (autonomic reflex testing) markers of SFN. Direct damage of sensory fibers (with abnormal ENFD only) was found in 90% of participants while 40% of participants had abnormalities of both sensory and autonomic (SGNFD) fibers. Therefore, SFN may be responsible for the pain experienced by patients with PTLDS.

Autonomic dysfunction of variable severity was found in all PTLDS participants. The dysautonomia was in most subjects mild to moderate. Orthostatic hypotension, a marker of more advanced autonomic failure, was detected in 1 subject only. The autonomic failure detected in this study may explain some of the symptoms experienced by PTLDS patients. Hence, this study suggests that autonomic testing may be useful in PTLDS subjects to provide an objective assessment of autonomic symptoms.

There are considerable controversies in definition and management of PTLDS [32,33]. Our study indicates that SFN may be an objective marker of PTLDS, at least in patients with prominent sensory symptoms. In addition, our findings suggest that quantitation of small fiber density and QASAT to grade severity of SFN and related dysautonomia may be useful endpoints in therapeutic trials.

Fatigue and cognitive complaints including “brain fog” are characteristic for PTLDS, yet their cause is unclear [8]. We found reduced CBFv in all PTLDS participants (70% PTLDS participants had abnormal orthostatic drop; the remaining 30% had low supine CBFv). Reduced CBFv in the context of absent systemic hypotension implies abnormal cerebral vasoconstriction and cerebral autoregulatory failure [36,37]. Cerebral vasoconstriction and abnormal cerebral vasoreactivity is associated with inflammation of cerebral vessels and poor cognitive functions in diabetes since inflammatory markers of endothelial integrity including soluble intercellular adhesion molecule (sICAM) and soluble vascular adhesion molecule (sVCAM) correlates with cerebral vasoconstriction [38,39]. Interestingly, both sICAM and sVCAM are elevated in Lyme disease [40]. T-cell chemokine CCL19, which is another inflammatory marker, is elevated during acute Lyme infection, and its elevation persists in PTLDS which may reflect ongoing inflammatory process [41]. We speculate that also elevation of sICAM and sVCAM persists in PTLDS and may result in inflammation-mediated cerebral vasoconstriction. Furthermore, reduced CBFv due to cerebral vasoconstriction in PTLDS can result in cerebral hypoperfusion as was already described in diabetes [39] or postural tachycardia syndrome (POTS) [42]. POTS patients also complaint of brain fog [43], similarly to PTLDS patients. An association between POTS and PTLDS has in fact been reported [44].

All our participants were treated with multiple antibiotics for prolonged time. Then the question arises if our findings, particularly the SFN, can be due to neurotoxic effect of medication. In randomized placebo-controlled PTLDS trials, where participants were treated with ceftriaxone and/or doxycycline, only the nonneurological antibiotics-related complications were common, affecting 25–43% of the participants. Most commonly reported where diarrhea, gastrointestinal bleeding, fever, anemia, allergic reactions and embolism. Significant neurotoxicity was not reported [10,44–46]. However, cephalosporins and penicillins can have central neurotoxic effect typically causing reversible encephalopathy [47], therefore in theory the antibiotics may cause brain fog which is poorly understood phenomenon and may be a type of cognitive complaint [43]. To our best knowledge, we did not find previous reports documenting chronic peripheral polyneuropathy as an effect of ceftriaxone, the second most common antibiotic used in our cohort. Reversible polyneuropathy due to doxycycline has been reported but it is very rare [48]. Therefore we concluded that it is unlikely that our results, particularly SFN, are due to toxic effects of antibiotics.

### Study limitations

This retrospective study has multiple limitations. The study has retrospective character and the studied group is small. A referral bias may affect selection of subjects. Patients with PTLDS in our cohort had prominent sensory complaints affecting limbs which prompted referral for testing and, therefore, the studied population may not be representative. Other biases may influence the results. The cerebral blood flow was assessed indirectly using CBFv. Although transcranial Doppler measures flow velocity instead of blood flow, CBFv is considered a good surrogate of CBF [49]. Nevertheless, dedicated cerebral perfusion studies may be used in future to assess cerebral perfusion directly in PTLDS. In addition, assessment of inflammatory markers may clarify their role in presumed inflammation of cerebral vessels. Although cognitive complaints are frequent in PTLDS, we did not perform formal cognitive evaluations in our cohort, therefore the link between low CBFv and cognitive impairment should be explored in future studies.

## Conclusions

SFN, related dysautonomia and abnormal CBFv appear to be associated with PTLDS. SFN may be responsible for sensory symptoms in PTLDS. Reduced orthostatic CBFv can be associated with cerebral hypoperfusion in PTLDS and may lead to cognitive dysfunction and brain fog. Autonomic failure detected in PTLDS is mild to moderate. These observations should be considered hypothesis generating and need to be confirmed by prospective controlled studies.

## Supporting information

S1 Table List of antibiotics used for treatment of Lyme disease. No, the participant number, the same as in Tables 1 and 3.
(DOCX)
