# Supplementary material for: Association of small fiber neuropathy and post treatment Lyme disease syndrome
Source: PLoS One. 2019 Feb 12;14(2):e0212222. doi: 10.1371/journal.pone.0212222 (PMC6372188; doi:10.1371/journal.pone.0212222)
Supplement: S1 Table — No, the participant number, the same as in Tables 1 and 3. (DOCX) [file pone.0212222.s001.docx]

| No | Antibiotic |
| --- | --- |
| 1 | Penicillin i.v. 24 million units/day x 1 month  Amoxicillin 500 mg po tid X 30 days  Clarithromycin  Atovaquone  Ceftriaxone i.v. x 41 days |
| 2 | Doxycyclin orally x 3 cycles,  Ceftriaxone, i.v. x 6 weeks |
| 3 | Doxycycline, i.v.  Ceftriaxone i.v, several months |
| 4 | Doxycycline x 1 month  Plaquenyl x 6 months  Biaxin x 6 months  Tetracyclin  Ceftriaxone i.v  Azythromycin i.v |
| 5 | Atovaquone  Doxycyclin x 10 months |
| 6 | Doxycycline,  Azithromycin  Hydrochloroquine  Rifampin,  Penicillin |
| 7 | Doxycyclin x 6 weeks  Ceftriaxone  Monocyclin  Nystatin |
| 8 | Doxycyclin x 2 months  Ceftriaxone i.v.,x 1 month, twice  Tigecycline, i.v.  Daptomycin x 3 months |
| 9 | Azithromycin  Atovaquone  Metronidazole  Doxycyclin p.o x 6 months |
| 10 | Ceftriaxone i.v. x 6 weeks |
